# A Practical Treatise on Diseases of the Skin

**Published:** 1893-04

**Authors:** 


A PRACTICAL TREATISE ON DISEASES OF THE SKIN—By John V. Shoemaker, A. M., M. D. Second edition, revised and enlarged. D, Appleton & Co., New York, 1892. Cloth $5.00, Sheep $6.00.

It gives us pleasure to review a work like the one before us. Dr. Shoemaker has handled the subject of skin diseases in a practical, concise way, and his book is well suited to the needs of the medical student, and general practitioner, as well as the specialist.

The work is divided into two parts. In Part I he treats in an interesting manner of the anatomy and physiology of the skin, and in a general way of the symptomatology, diagnosis, pathology, etiology and treatment of skin diseases. He gives a few anatomical plates of the skin, which are plain and accurate.

In Part II he deals with the individual diseases in a readable and instructive style. He does not, as most writers on this subject are inclined to do, elaborate the nomenclature to the bewilderment of many readers. His classification is a modification of Hebra's, made upon an anatomical and pathological basis.

He gives a few photo-micrographs, and wood cut, which add to the value of the work.

The results of the treatment of lupus by tuberculine are given, which the author claims is less satisfactory than by the old methods.

Quite a number of formulas have been embodied in this edition, which, it is claimed, have been tried and not found wanting.

The book is handsomely gotten up, and well bound, and should be in the library of every physician- J. A. C.

